# Retraction for Uddén et al., “A Nonfunctional Opsonic Antibody Response Frequently Occurs after Pneumococcal Pneumonia and Is Associated with Invasive Disease”

**DOI:** 10.1128/mSphere.01101-20

**Published:** 2020-12-16

**Authors:** Fabian Uddén, Jonas Ahl, Nils Littorin, Kristoffer Strålin, Simon Athlin, Kristian Riesbeck

**Affiliations:** a Clinical Microbiology, Department of Translational Medicine, Faculty of Medicine, Lund University, Malmö, Sweden; b Infectious Diseases, Department of Translational Medicine, Faculty of Medicine, Lund University, Malmö, Sweden; c Unit of Infectious Diseases, Department of Medicine, Huddinge, Karolinska Institutet, Stockholm, Sweden; d Department of Infectious Diseases, Faculty of Medicine and Health, Örebro University, Örebro, Sweden

## RETRACTION

Volume 5, no. 1, e00925-19, 2020, https://doi.org/10.1128/mSphere.00925-19. We hereby retract this article published in *mSphere*. In this paper, we presented a study of the adaptive immune response of patients with pneumococcal community-acquired pneumonia based on a serotype-specific opsonophagocytic assay (OPA) with paired acute-phase and convalescent-phase sera. A reduced function of the convalescent-phase sera compared to that of the acute-phase sera or undetectable OPA titers in both sera was detected in 35% of cases. These responses that were assessed as nonfunctional were significantly associated with bacteremia. Outcomes in the OPA were also compared with previously determined concentrations of serotype-specific anticapsular Ig in the sera, and we did not detect any significant associations in these analyses.

Unfortunately, we recently discovered that incorrect data were used in the analyses of anticapsular Ig concentrations that are presented in the published text. The numbers used were geometric mean concentrations of anticapsular Ig to six different pneumococcal serotypes, not serotype-specific Ig which was our intention and what was stated in the paper. Due to this error, certain conclusions presented in the paper regarding the comparisons of OPA titers and anticapsular Ig concentrations are not valid as they are based on unrelated data.

Importantly, the main conclusions of the study, related to the results from the OPA, are still valid, i.e., an episode of pneumococcal pneumonia is often an immunizing event, resulting in an improved serotype-specific adaptive immune status as measured with OPA, but a nonfunctional antibody response may occur and is significantly associated with bacteremia. For the above-mentioned reason, we would like to retract our article, and we apologize for the inconvenience it may have caused to the readers. We intend to submit a revised version of the manuscript presenting the corrected analyses that provided statistically significant results and more comprehensive conclusions.

